# Tocilizumab for cystoid macular edema secondary to retinitis pigmentosa

**DOI:** 10.1186/s12348-024-00430-0

**Published:** 2024-09-30

**Authors:** Stéphane Abramowicz, Prochore Kamgang Semeu, Isabelle Nubourgh, Laurence Postelmans, François Willermain

**Affiliations:** 1 Department of Ophthalmology, Saint-Pierre University Hospital, Université Libre de Bruxelles, Brussels, Belgium; 2 Department of Ophthalmology, Brugmann University Hospital, Université Libre de Bruxelles, Brussels, Belgium; 3 Department of Internal Medicine, Brugmann University Hospital, Université Libre de Bruxelles, Brussels, Belgium; 4 Department of Internal Medicine, Saint-Pierre University Hospital, Université Libre de Bruxelles, Brussels, Belgium

**Keywords:** Cystoid macular edema, Posterior uveitis, Retinitis pigmentosa, Tocilizumab, Uveitis masquerade syndrome

## Abstract

**Purpose:**

To describe the effect of tocilizumab (TCZ) on cystoid macular edema (CME) and retinal vascular leakage (RVL) in retinitis pigmentosa (RP).

**Methods:**

Retrospective case series.

**Results:**

We present 2 cases of RP with marked inflammatory features in the form of CME and RVL. There was initial diagnostic uncertainty with posterior uveitis. Both patients were treated with corticosteroids, conventional disease-modifying antirheumatic drugs (cDMARDs), and biological DMARDs (bDMARDs) for the inflammatory features with partial and inconsistent treatment response. When treatment was switched to intravenous (IV) TCZ, dramatic reduction in CME and RVL were observed in both patients. Diagnosis of RP was eventually made based on findings of ancillary tests (macular spectral-domain optical coherence tomography, visual fields, full-field electroretinogram). Genetic testing led to a molecular diagnosis of *EYS*-related autosomal recessive RP in patient 1, while patient 2 had negative gene panel results.

**Conclusions:**

IV TCZ can be an effective treatment option in RP-related CME and RVL. Whether this treatment strategy has an effect on prognosis remains to be established, but it is possible considering chronic CME-related retinal damage is a major driver of central vision loss in RP.

## Background

Tocilizumab (TCZ) is a humanized monoclonal antibody targeting soluble and membrane-bound interleukin 6 receptors (IL-6R). TCZ has proven efficacy in a wide range of immune-mediated inflammatory diseases (IMIDs) such as systemic juvenile idiopathic arthritis, rheumatoid arthritis, and giant cell arteritis [1–3]. Interleukin 6 (IL-6) blockade has also been used for uveitic macular edema (UME), mainly in refractory cases [4–6]. 

IL-6 is a proinflammatory cytokine with pleiotropic effects, including promoting the differentiation of IL-17-producing T helper (Th17) cells, and blocking the generation and activity of T regulator (Treg) cells. Hence, IL-6 is considered a regulator of Treg/Th17 balance, inducing lower immunologic tolerance and promoting the development of IMIDs, including autoimmune uveitis [7]. Besides the inflammatory route, IL-6 can induce macular edema (ME) through other pathways such as production of vascular endothelial growth factor (VEGF) and downregulation of tight junction proteins in retinal endothelial cells leading to retinal vascular leakage (RVL) [7]. 

IL-6 blockade has also proven useful for ME arising from other causes, such as pseudophakic ME (PME) or ME secondary to non-paraneoplastic autoimmune retinopathy (npAIR) [7–9]. 

There has been a single other report on intravenous (IV) TCZ administration for retinitis pigmentosa (RP)-related cystoid macular edema (CME) [10]. We herein report the second series of patients treated with IV TCZ for RP-related CME and RVL.

## Methods

### Study design

Retrospective case series.

### Ethics approval

The research adhered to the tenets set forth in the Declaration of Helsinki and was approved by our local ethics committee (CE 2023/157). Written informed consent for publication was obtained from both patients.

### Case descriptions

#### Case 1

An 18-year-old man complained of visual field defects, mostly at nighttime, that started during childhood. There was no notable general or family history. He was of Moroccan ethnicity, and was not taking any medication.

**Fig. 1:**
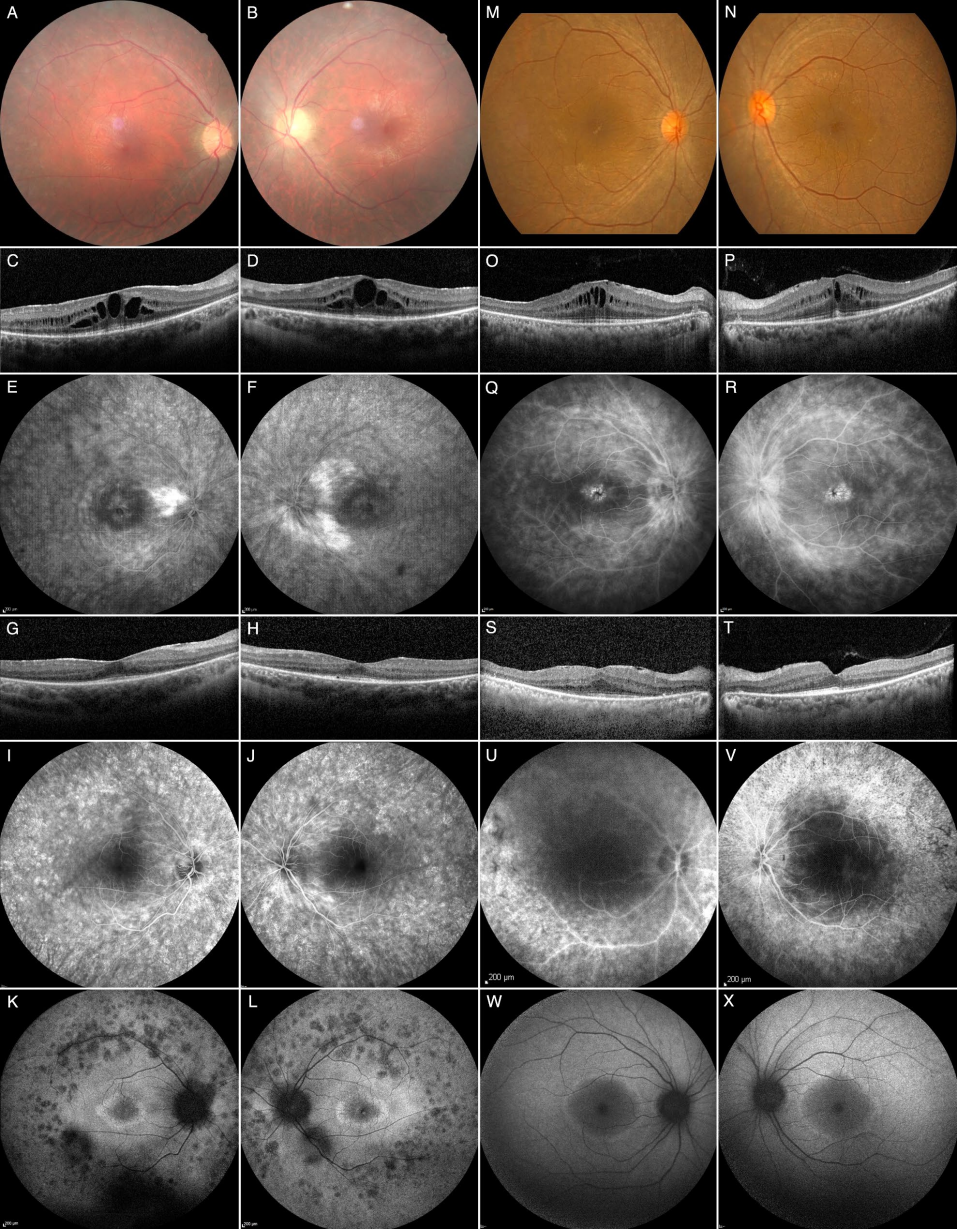
Retinal findings before and after treatment with intravenous tocilizumab in patients 1 and 2. **Patient 1**, ***EYS*-related autosomal recessive retinitis pigmentosa – A**, Fundoscopy of the right eye showing mild disc hyperemia, cellophane maculopathy, vascular attenuation, and mid-peripheral outer retinal atrophy (ORA). **B**, Fundoscopy of the left eye showing similar findings except for mild temporal disc pallor. **C – D**, Before treatment with intravenous tocilizumab (IV TCZ), macular spectral-domain optical coherence tomography (SD-OCT) shows an epiretinal membrane, cystic changes in the outer (ONL) and inner nuclear layers (INL), some very large at the level of the fovea, and perifoveal ORA in both eyes. **E – F**, Before treatment with IV TCZ, fluorescein angiogram (FA) shows intense peripapillary retinal capillaritis, foveal leakage, and patchy hyperfluorescent defects over the posterior pole and beyond the vascular arcades in both eyes. **G – H**, Macular SD-OCT showing perifoveal ORA, and complete resolution of the cystic changes in both eyes after treatment with IV TCZ. **I – J**, FA shows generalized patchy window defects over the posterior pole and beyond the vascular arcades, and mild capillary leakage along the proximal temporal vascular arcades in both eyes. Note the substantial reduction in vascular leakage after treatment with IV TCZ when compared with pre-treatment images. **K – L**, Blue light autofluorescence (BAF) shows a perifoveal hyperautofluorescent ring and patchy mid-peripheral hypoautofluorescence in both eyes. **Patient 2, RP without molecular explanation*–*M – N**, Fundoscopy showing mild optic disc hyperemia, blunted foveal reflex, subtle mid-peripheral ORA and mild vascular attenuation in both eyes. **O – P**, Before treatment with IV TCZ, macular SD-OCT shows an epiretinal membrane, cystic changes predominantly in the INL, and perifoveal ORA in both eyes. **Q – R**, Before treatment with IV TCZ, FA shows severe multifocal leakage from the optic disc, fovea, capillaries, and veins in both eyes. **S – T**, Macular SD-OCT showing perifoveal ORA, and dramatic reduction of the cystic changes in both eyes after treatment with IV TCZ. **U – V**, After treatment with IV TCZ, FA shows patchy mid-peripheral window defects extending beyond the vascular arcades in both eyes. Some newly occurring hypofluorescent lesions secondary to intraretinal pigment migrations are visible in the temporal mid-periphery. Note the dramatic reduction in vascular leakage after treatment with IV TCZ when compared with pre-treatment images. **W – X**, BAF shows central hypoautofluorescence surrounded by a discrete perifoveal hyperautofluorescent ring in both eyes

On examination, best-corrected visual acuity (BCVA) was 20/32 in both eyes (OU). Slit-lamp examination (SLE) was normal except for 1 + anterior vitreous cells. Fundoscopy showed vascular attenuation and mid-peripheral outer retinal atrophy (ORA) (Fig. 1, A – B). There were no intraretinal pigment migrations (IPMs). Macular spectral-domain optical coherence tomography (SD-OCT) showed CME and perifoveal ORA (Fig. 1, C – D). Fluorescein angiography (FA) demonstrated peripapillary leakage and CME (Fig. 1, E – F). Humphrey 24 − 2 visual fields (HVF) showed pericentral sensitivity loss. Full-field electroretinogram (ffERG) was uninterpretable because of contact lens electrode aversion.

Posterior uveitis (PU) in the form of peripapillary retinal capillaritis was suspected and a targeted uveitis work-up (syphilis, tuberculosis, sarcoidosis, Birdshot retinochoroiditis [BRC]) was negative. Owing to perifoveal ORA, RP masquerading as PU was then suspected and an inherited retinal disease (IRD) gene panel was ordered.

Treatment for waxing and waning CME was initiated with partial and inconsistent response. This included topical diclofenac sodium 0.1% bid, topical brinzolamide 1% tid, oral methylprednisolone, oral methotrexate (MTX), anti-VEGF intravitreal injections (IVT), MTX IVT, dexamethasone implant (DEX) IVT, and intravenous (IV) infliximab, before obtaining CME and RVL control using IV TCZ 8 mg/kg every 4 weeks (Fig. 1, G – J). It took approximately 2 to 3 weeks to obtain CME and RVL control with TCZ.

Ultimately, a homozygous pathogenic variant in the *EYS* gene was found and a diagnosis of *EYS*-related autosomal recessive RP was made. Scarce peripheral IPMs had appeared over the follow-up.

Comprehensive explanations were given to the patient concerning the genetic nature of the disease, and the absence of a specific treatment. However, considering the obvious benefit of TCZ on the inflammatory component of the disease, the patient opted to pursue treatment.

At the last follow-up, macular SD-OCT remained dry under IV TCZ 8 mg/kg every 8 weeks. Final BCVA was 20/25 OU. Total follow-up was 129 months, of which 103 were under treatment with TCZ.

#### Case 2

A 61-year-old woman presented with nyctalopia, difficulty stumbling over things, and photopsia for the past year. She was of Chinese ethnicity, and had a history of cysticercosis for which she had been inconsistently self-medicating with praziquantel.

On examination, BCVA was 20/30 in the right eye, and 20/25 in the left. SLE was normal. Fundus examination revealed subtle mid-peripheral ORA, and mild vascular attenuation (Fig. 1, M – N). Macular SD-OCT showed symmetrical perifoveal ORA, and CME (Fig. 1, O – P). FA revealed intense vascular leakage from the optic disc, veins, capillaries, and fovea (Fig. 1, Q – R). HVF showed concentric defects. ffERG revealed rod-cone dysfunction (RCD). Overall, findings were felt to be compatible with RP and an IRD gene panel was ordered.

However, owing to intense CME and RVL, a targeted uveitis (syphilis, tuberculosis, sarcoidosis, BRC, cysticercosis) work-up was performed, which was negative, and treatment with topical prednisolone acetate 1% tid, topical ketorolac trometamol 0.5% tid, and 0.8 mg/kg oral methylprednisolone was initiated. This treatment yielded no benefit after 1 month and was discontinued.

Over the ensuing months, BCVA dropped further to 20/50 in the right eye, and 20/32 in the left. Gene panel results showed a heterozygous pathogenic variant in the *KCNV2* gene, and a variant of unknown significance in the *TSPAN12* gene. These findings did not help establish a molecular diagnosis of RP. Scarce perivascular IPMs had appeared in the retinal periphery. Given the absence of RVL and CME improvement with corticosteroids, alongside compatible ancillary examination findings, a formal clinical diagnosis of RP was made.

The dual nature of the disease was explained to the patient (RP with inflammatory features, or possible associated non-infectious PU), and she agreed to a stepladder immunosuppressive approach.

Treatment for CME and RVL was then initiated with inconsistent responses. This included topical brinzolamide 1% tid, topical ketorolac trometamol 0.5% tid, oral methylprednisolone, oral MTX, DEX IVT, and subcutaneous adalimumab, before ultimately obtaining CME and RVL control using IV TCZ 8 mg/kg every 4 weeks (Fig. 1, S – V). It took approximately 2 to 3 weeks to obtain CME and RVL control with TCZ.

At the last follow-up, macular SD-OCT remained dry under IV TCZ 8 mg/kg every 4 weeks. Final BCVA was 20/32 in the right eye, and 20/40 in the left. Total follow-up was 63 months, of which 19 were under treatment with TCZ.

## Discussion

IL-6 blockade with TCZ is effective in controlling ME of various etiologies like UME, PME, and ME secondary to npAIR [4, 5, 7–10]. 

In this short cases series, we demonstrate the excellent effect of TCZ on RP-related CME and RVL. These data are consistent with the recent case series described by Méndez-Martínez et al. [10] This is also in keeping with the finding that IL-6 plays a critical role in the genesis of ME [7], and that IL-6, alongside several other pro-inflammatory cytokines, is up-regulated in patients with RP [11]. Therefore, we can reasonably assume that IL-6 pathway activation is a mechanistic component of RP-related CME and RVL. Presence of an inflammatory response in RP has been well-documented, and it is thought to be secondary to forms of regulated photoreceptor necrosis, e.g. necroptosis or ferroptosis [12]. Moreover, it has been shown that patients with RP who have more severe vitreous inflammation have significantly lower visual function [11]. This evidence suggests a role for anti-inflammatory drugs in a subset of RP patients with marked inflammatory features, for which we propose the term inflammatory RP (iRP).

Both patients were evaluated for PU because of presence of significant CME and RVL, ultimately justifying the choice of treatment with IV TCZ. However, it became apparent during the follow-up that they suffered from iRP. Indeed, hereditary ocular disorders, including RP, are a well-known type of uveitis masquerade syndrome (UMS), accounting for up to 31% of non-neoplastic UMS [13]. iRP cases are those in which CME is severe, vascular leakage on FA is intense, and vitreous cells are present.

Another diagnostic confounder for these entities is the possibility of RP sine pigmento. Takahashi et al. reported on a cohort of 240 RP patients, 213 (89%) of which had IPMs at the time of RP diagnosis. They found bone spicule formation in 10/27 (37%) RP sine pigmento patients, with a median time to appearance of bone spicules of 5.4 years from the first visit [14]. Thus, RP sine pigmento probably occur rarely, and many RP sine pigmento patients do develop pigment deposition over time.

The main signs to distinguish iRP from idiopathic PU presenting as retinal vasculitis and CME are the presence of symmetrical perifoveal ORA on macular SD-OCT, and symmetrical hyperautofluorescent perifoveal rings on blue light autofluorescence. These would be expected in RP but not uveitis [15]. Indeed, for ORA to develop from uveitis, the choroid/choriocapillaris or retinal pigment epithelium must be involved in order to either compromise outer retinal perfusion or hamper photoreceptor homeostasis, respectively. Post-inflammatory ORA is frequently seen as a result of uveitis, but is usually distributed in a haphazard, random fashion, corresponding to where inflammatory foci lay [15]. Pericentral scotomas, nyctalopia, and RCD are additional diagnostic clues for RP, not typically expected in uveitis [15]. 

iRP are ophthalmological examples of genetic diseases exhibiting marked inflammatory features. As such, their optimal treatment strategy might need to involve both gene therapy, and anti-inflammatory drugs, simultaneously. To date, it has not been demonstrated that treating iRP with TCZ leads to a better visual prognosis. However, considering chronic CME leads to worsening of macular function over time, one would instinctively assume that this could be beneficial. Obviously, side effects of any new treatment must be taken into account and discussed with patients accordingly. Nevertheless, preserving macular photoreceptors and maintaining adequate central vision for RP patients should remain our primary focus.

## Conclusions

IV TCZ can be an effective treatment option in RP-related CME and RVL. Whether this treatment strategy has an effect on prognosis remains to be established.

## Data Availability

No datasets were generated or analysed during the current study.
